# Mitochondrial targets in ischaemic heart disease and heart failure, and their potential for a more efficient clinical translation. A scientific statement of the ESC Working Group on Cellular Biology of the Heart and the ESC Working Group on Myocardial Function

**DOI:** 10.1002/ejhf.3674

**Published:** 2025-05-04

**Authors:** Melanie Paillard, Mahmoud Abdellatif, Ioanna Andreadou, Christian Bär, Luc Bertrand, Bianca J.J.M. Brundel, Gemma Chiva‐Blanch, Sean M. Davidson, Dana Dawson, Fabio Di Lisa, Paul Evans, Zoltan Giricz, Derek J. Hausenloy, Petra Kleinbongard, Frank Lezoualc'h, Elisa Liehn, Christoph Maack, Ange Maguy, Elizabeth Murphy, Cinzia Perrino, Maurizio Pesce, Peter P. Rainer, Katrin Streckfuss‐Bömeke, Matthias Thielmann, Rong Tian, Carlo G. Tocchetti, Jolanda Van Der Velden, Sophie Van Linthout, Serena Zacchigna, Thomas Krieg

**Affiliations:** ^1^ Laboratoire CarMeN‐IRIS Team, INSERM, INRA, Université Claude Bernard Lyon‐1 Bron France; ^2^ Division of Cardiology, Department of Internal Medicine Medical University of Graz, BioTechMed Graz Graz Austria; ^3^ Laboratory of Pharmacology, School of Pharmacy, National and Kapodistrian University of Athens Athens Greece; ^4^ Institute of Molecular and Translational Therapeutic Strategies (IMTTS), Hannover Medical School, Germany‐Fraunhofer Institute for Toxicology and Experimental Medicine (ITEM) Hannover Germany; ^5^ UCLouvain, Institute for Experimental and Clinical Research, Pole of Cardiovascular Research; WELBIO Department, WEL Research Institute Brussels Belgium; ^6^ Physiology, Amsterdam UMC Location Vrije Universiteit, Cardiovascular Sciences, Heart Failure and Arrhythmias Amsterdam The Netherlands; ^7^ Faculty of Health Sciences, Universitat Oberta de Catalunya, Department of Endocrinology and Nutrition, August Pi i Sunyer Biomedical Research Institute (IDIBAPS) Hospital Clínic of Barcelona Barcelona Spain; ^8^ Biomedical Network Research Centre on Obesity and Nutrition Physiopathology (CIBEROBN), Instituto de Salud Carlos III Madrid Spain; ^9^ The Hatter Cardiovascular Institute, University College London London UK; ^10^ Aberdeen Cardiovascular and Diabetes Centre, School of Medicine and Dentistry, University of Aberdeen Aberdeen UK; ^11^ Department of Biomedical Sciences, University of Padua Padova Italy; ^12^ William Harvey Research Institute, Queen Mary University of London London UK; ^13^ Department of Pharmacology and Pharmacotherapy Semmelweis University Budapest Hungary; ^14^ National Heart Research Institute Singapore, National Heart Centre Singapore Singapore Singapore; ^15^ Cardiovascular and Metabolic Disorders Program, Duke‐National University of Singapore Medical School Singapore Singapore; ^16^ Yong Loo Lin School of Medicine, National University Singapore Singapore Singapore; ^17^ Institute of Pathophysiology, Faculty of Medicine University of Duisburg‐Essen Duisburg Germany; ^18^ Institute of Metabolic and Cardiovascular Diseases (I2MC), INSERM, University of Toulouse III – Paul Sabatier Toulouse France; ^19^ Institute for Molecular Medicine University of Southern Denmark Odense Denmark; ^20^ Department of Translational Research Comprehensive Heart Failure Center (CHFC), and Medical Clinic I, University Clinic Würzburg Würzburg Germany; ^21^ Atrial Fibrillation and Therapeutic Innovation, Department of Physiology Faculty of Medicine, University of Bern Bern Switzerland; ^22^ Cardiac Physiology, NHLBI, NIH Bethesda MD USA; ^23^ Department of Advanced Biomedical Sciences Federico II University Naples Italy; ^24^ Unit of Cardiovascular Tissue Engineering Centro Cardiologico Monzino, IRCCS Milan Italy; ^25^ Department of Cell Biology King Faisal Specialist Hospital & Research Center Riyadh Saudi Arabia; ^26^ Department of Mechanical and Aerospace Engineering (DIMEAS) Turin School of Engineering Turin Italy; ^27^ Institute of Pharmacology and Toxicology, University of Würzburg, Germany‐Clinic for Cardiology and Pneumology, University Medicine Göttingen, and German Center for Cardiovascular Research (DZHK), Partner site Göttingen Göttingen Germany; ^28^ West‐German Heart & Vascular Center, University Duisburg‐Essen Essen Germany; ^29^ Mitochondria and Metabolism Center, Department of Anesthesiology & Pain Medicine University of Washington Seattle WA USA; ^30^ Department of Translational Medical Sciences Center for Basic and Clinical Immunology Research, Interdepartmental Center of Clinical and Translational Sciences, Interdepartmental Hypertension Research Center, Federico II University Naples Italy; ^31^ Department of Physiology Amsterdam UMC, Vrije Universiteit Amsterdam, The Netherlands‐Amsterdam Cardiovascular Sciences, Heart Failure & Arrhythmias Amsterdam The Netherlands; ^32^ Berlin Institute of Health (BIH) at Charité, Universitätmedizin Berlin, Germany, BIH Center for Regenerative Therapies (BCRT), German Center for Cardiovascular Research (DZHK), Partner site Berlin Berlin Germany; ^33^ Laboratory of Cardiovascular Biology, The International Centre for Genetic Engineering and Biotechnology, Department of Medicine, Surgery and Health Sciences University of Trieste Trieste Italy; ^34^ Department of Medicine University of Cambridge Cambridge UK

**Keywords:** Ischaemia–reperfusion injury, Heart failure, Mitochondria‐targeted drug therapy, Cardioprotection

## Abstract

Acute myocardial infarction (MI) remains a major cause of death and disability worldwide. No adjuvant treatment has yet been fully validated in patients to limit the progression from the initial tissue damage due to acute MI, to the development of heart failure. However, mitochondria have long been demonstrated to be a key target for cardioprotective strategies to reduce cell death that leads to left ventricular dysfunction and ultimately heart failure. While pre‐clinical studies have investigated several mitoprotective strategies targeting different mitochondrial functions, such as oxidative stress or permeability transition pore opening, none have shown successful clinical translation so far. In this European Society of Cardiology scientific statement, we present recent research advances in the understanding of the mitochondrial alterations occurring in MI and in the discovery of key components of mitochondrial structure and function in order to improve drug development. We discuss the reasons for the failure of clinical translation and the remaining obstacles that need to be addressed, including timing of drug administration, tissue bioavailability and efficient mitochondrial targeting, together with the mitochondrial impact derived from risk factors, comorbidities and comedications. Taken together, this scientific statement aims to provides a consensus opinion from clinicians and basic scientists to translate some of the most promising mitoprotective targets into the clinical setting to protect against MI and heart failure.

## Introduction

**Figure 1:**
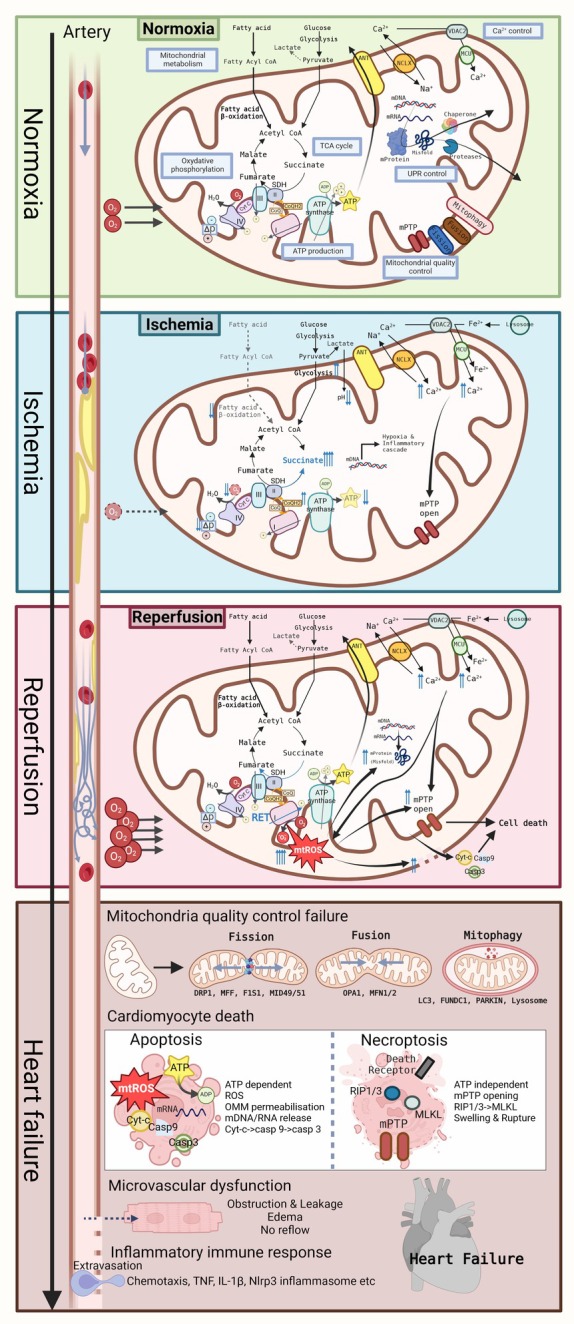
Evolution of mitochondrial dysfunction during the progression of ischaemic heart disease. Hypothetical sequence of mitochondrial events during ischaemia/reperfusion and subsequent development of heart failure. ADP, adenosine diphosphate; ANT, adenine nucleotide translocator; ATP, adenosine triphosphate; Casp3, caspase‐3; Casp9, caspase‐9; CoA, coenzyme A; Cyt‐c, cytochrome C; DRP1, dynamin‐related protein 1; F1S1, mitochondrial fission protein 1; FUNDC1, FUN14 domain‐containing protein 1; IL, interleukin; LC3, microtubule‐associated protein‐1 light chain‐3; MCU, mitochondrial calcium uniporter; MFF, mitochondrial fission factor; MFN1, mitofusin 1; MFN2, mitofusin 2; MID49/51, mitochondrial dynamics proteins of 49/51 kDa; MLKL, mixed‐lineage kinase domain‐like pseudokinase; mPTP, mitochondrial permeability transition pore; mtROS, mitochondrial reactive oxygen species; NCLX, Na^+^/Ca^2+^ exchanger; Nrlp3, nucleotide‐binding oligomerization domain‐like receptor family, pyrin domain containing protein 3; OMM, outer mitochondrial membrane; OPA1, optic atrophy 1; RET, reverse electron transport; RIP1/3, receptor‐interacting protein 1/3; SDH, succinate dehydrogenase; TCA, tricarboxylic acid; TNF, tumour necrosis factor; UPR, unfolded protein response; VDAC, voltage‐gated anion channel.

Myocardial infarction (MI) remains one of the leading causes of death and disability worldwide. Following the acute phase of myocardial ischemia–reperfusion (I/R) which occurs during MI, patients often develop adverse myocardial remodelling, ischaemic cardiomyopathy and heart failure (HF). Although post‐MI mortality has reduced over the last decades, there is still no treatment for preventing the deleterious progression of MI.^1^ Both pre‐clinical and clinical studies have shown the crucial role of mitochondria as a target for cardioprotection. Through their various functions, mitochondria represent a key regulator of cell fate. Importantly, the MI‐associated mitochondrial dysfunction changes with the progression of the disease (*Figure* *1*). For example, mitochondrial Ca^2+^ overload occurs during the acute phase of cardiac I/R^2^ while a lower mitochondrial Ca^2+^ content is measured in the failing heart.^3^ Similarly, oxidative stress with mitochondrial reactive oxygen species (ROS) being excessively produced during I/R is detrimental but has also been shown to exert critical protective roles in the subsequent remodelling process. Therefore, deciphering the chronology of mitochondrial dysfunction in the progression of MI to HF is crucial to adapt treatments targeting mitochondrial function.

In the present scientific statement, we aim to highlight the importance of targeting specific mitochondrial functions at defined times to ameliorate ischaemic heart disease (IHD), from I/R injury to HF. We explain how the improvements in the understanding of the mitochondrial alterations that occur during MI have led to the development of new, more promising treatments. We will also discuss the challenges in translating these strategies from experimental models to patients, taking into consideration sex, age, comorbidities, concurrent medications, and other potential cellular targets.

This scientific statement will focus on the cardiomyocyte and treatments targeted at its survival. It should however be highlighted that many other cardiac cell types and structures are involved in the discussed process during I/R injury, e.g. coronary vascular injury, which is reviewed elsewhere.^4^, ^5^

## Mitochondria as a therapeutic target in myocardial infarction and heart failure: pre‐clinical and clinical evidence

During I/R, disruption of mitochondrial function leads to increased ROS formation and impaired Ca^2+^ homeostasis. Consequently, the apoptosis and necrosis cell death pathways are initiated with the opening of the mitochondrial permeability transition pore (PTP). The initial development of mitochondrially targeted therapeutic strategies against MI have therefore mainly focused on limiting mitochondrial ROS production and PTP activation. However, while Phase 2 clinical trials were encouraging, all larger clinical trials targeting this avenue failed to show cardioprotective benefits (*Table* *1*),^6^, ^7^, ^8^, ^9^, ^10^, ^11^, ^12^, ^13^, ^14^, ^15^, ^16^, ^17^, ^18^, ^19^, ^20^, ^21^, ^22^, ^23^, ^24^, ^25^ calling for additional mitochondrial targets, and more refined and novel approaches as discussed below.

**Table 1:** Clinical trials investigating mitochondrial treatments for the treatment of ischaemic heart disease

Author	Study design	Treatment	Primary endpoint	Results
**Oxidative stress**				
Gibson *et al*., 2016^6^	EMBRACE STEMI: Phase 2a, 118 STEMI patients	Elamipretide/MTP‐131: a cell‐permeable peptide that preserves the integrity of cardiolipin Infusion for 1 h	Infarct size by CK AUC	Safe and well‐tolerated administration No reduction in infarct size
Dominguez‐Rodriguez *et al*., 2017^7^	MARIA: 146 STEMI patients	Melatonin Intravenous and intracoronary injection	Infarct size by MRI	No reduction in infarct size Unfavourable effect on LV function
Mortensen *et al*., 2014^8^	Q‐SYMBIO: 420 patients with moderate to severe HF	CoQ10: powerful antioxidant Given at a dose of 100 mg 3 times daily for 2 years	Short‐term: NYHA classification Long‐term: MACEs at week 106: time to first event	No change in short‐term endpoint Significantly fewer MACEs at 2 years
Daubert *et al*., 2017^9^	36 HFrEF patients	A single 4‐h infusion of elamipretide	Safety and efficacy up to 24 h post‐infusion	Safe and well‐tolerated administration Favourable significant decreased LVEDV
Butler *et al*., 2020^10^	71 HFrEF patients	4 mg or 40 mg elamipretide once daily by subcutaneous injection for 28 consecutive days	Change in LVESV from baseline evaluated by cardiac MRI	Elamipretide well tolerated No improvement of LVESV
Gal *et al*., 2020^11^	60 HFrEF patients	Resveratrol: antioxidant Given at a dose of 100 mg (capsules) daily for 3 months	Echocardiography, a 6‐min walk test, spirometry, quality of life questionnaire, lab test and RNA profile analysis	Improved systolic and diastolic LV function Improved quality of life and exercise capacity Decreased NT‐proBNP and galectin‐3 levels
**Mitochondrial bioenergetics**				
Lee *et al*., 2005^12^	56 CHF patients	Perhexiline: blocker of muscle mitochondrial free fatty acid uptake for 8 weeks	Peak exercise VO_2_	Improved VO_2_ max and LVEF
Holubarsch *et al*., 2007^13^	ERGO: 350 CHF patients planned, but early termination: 51 patients completed the study	Etomoxir: inhibitor of mitochondrial CPT1 Given at a dose of 80 or 40 mg for 6 months	A maximal exercise tolerance test and a submaximal 6‐min corridor walk test	Due to early termination for hepatic toxicity of etomoxir, not enough patients included to conclude
Fragasso *et al*., 2006^14^	55 HF patients	Trimetazidine: a fatty acid oxidation inhibitor Given at a dose of 20 mg 3 times daily for 1 year	NYHA functional class and EF	Improved NYHA class and EF after 1 year
Fragasso *et al*., 2006^15^	12 HF patients	Cross‐over study to placebo or trimetazidine (20 mg 3 times daily) for two periods of 90 days	NYHA/EF, PCr/ATP ratio by cardiac ^31^P‐MRS	Improved NYHA and EF associated with an increased PCr/ATP ratio
van de Bovenkamp *et al*., 2023^16^	DoPING‐HFpEF: Phase 2, 25 HFpEF patients	Trimetazidine treatment: pills 3 times per day or placebo, for 3 months and switched after a 2‐week wash‐out period	Change in pulmonary capillary wedge pressure	No improvement of myocardial energy homeostasis and exercise haemodynamics
Zhao *et al*., 2020^17^	246 HF patients caused by coronary heart diseases	Levocarnitine: free‐fatty acid transport into mitochondria Daily intravenous injection of 3.0 g for 2 weeks	Automatic biochemical analyser: levels of ALB, hs‐CRP, BNP, and troponin	Improved ALB, hs‐CRP, BNP, troponin, and LVDD levels
**PTP and cell death**				
Piot *et al*., 2008^18^	58 STEMI patients	Cyclosporin: PTP inhibitor An intravenous bolus of 2.5 mg/kg before PCI	Infarct size by CK and TnI release	Significant smaller infarct
Mewton *et al*., 2010^19^	28 STEMI patients	Cyclosporin An intravenous bolus at 2.5 mg/kg before PCI	Cardiac MRI	Persistence of reduced infarct size: reduction of LVESV
Ghaffari *et al*., 2013^20^	101 STEMI patients	Cyclosporin An intravenous bolus injection at 2.5 mg/kg immediately before TLT	Occurrence of MACEs	No reduction in infarct size and no improvement in MACEs
Cung *et al*., 2015^21^	CIRCUS: 970 STEMI patients	Cyclosporin An intravenous bolus injection before coronary recanalization	MACEs	No improvement in MACEs
Ottani *et al*., 2016^22^	CYCLE: Phase 2, 410 STEMI patients	Cyclosporin An intravenous bolus at 2.5 mg/kg before PCI	Incidence of ≥70% ST‐segment resolution 60 min after TIMI flow grade 3	No effect on ST‐segment resolution or hs‐cTnT
Atar *et al*., 2015^23^	MITOCARE: 163 STEMI patients	TRO40303: PTP inhibitor An intravenous bolus injection prior to balloon inflation during PCI	Infarct size by CK and TnI AUC	No reduction in CK and TnI AUC Increased adjudicated safety events

^31^P‐MRS, phosphorous magnetic resonance spectroscopy; ALB, albumin; ATP, adenosine triphosphate; AUC, area under the curve; BNP, B‐type natriuretic peptide; CHF, chronic heart failure; CK, creatine kinase; CoQ10, coenzyme Q10; CPT1, carnitine palmitoyltransferase 1; EF, ejection fraction; HF, heart failure; HFpEF, heart failure with preserved ejection fraction; HFrEF, heart failure with reduced ejection fraction; hs‐CRP, high‐sensitivity C‐reactive protein; hs‐cTnT, high‐sensitivity cardiac troponin T; LVEDV, left ventricular end‐diastolic volume; LV, left ventricular; LVDD, left ventricular end‐diastolic dimension; LVEF, left ventricular ejection fraction; LVESV, left ventricular end‐systolic volume; MACE, major adverse cardiovascular event; MRI, magnetic resonance imaging; NT‐proBNP, N‐terminal pro‐B‐type natriuretic peptide; NYHA, New York Heart Association; PCI, percutaneous coronary intervention; PCr, phosphocreatine; PTP, permeability transition pore; STEMI, ST‐elevation myocardial infarction; TIMI, Thrombolysis in Myocardial Infarction; TLT, thrombolytic treatment; TnI, troponin I; VO_2_, oxygen consumption.

### Oxidative stress

Increased ROS due to redox imbalance has been widely evidenced in diverse ischaemic cardiac pathologies using various model systems.^26^, ^27^, ^28^, ^29^ Despite the intensive research in the field, so far, no drug targeting mitochondrial ROS or mitochondrial antioxidant systems during I/R has been shown to be clinically effective. Therefore, there is an evident need to develop novel efficacious and safe medications to alleviate mitochondrial oxidative stress in IHD (*Figure* *1*).

#### Inhibiting reactive oxygen species production

Mitochondria are the leading source of ROS during I/R. It is now well‐established that during ischaemia, the tricarboxylic acid cycle metabolite succinate accumulates and on reperfusion, this drives a highly specific mechanism of reverse electron transport (RET) at complex I.^30^ The succinate which is responsible for the RET‐ROS mechanism is oxidized by succinate dehydrogenase (SDH), and recent evidence has shown that short‐term SDH inhibition is highly protective against I/R injury and HF.^31^, ^32^ Furthermore, the most‐suitable SDH inhibitor, malonate, is preferably taken up by ischaemic tissue due to the low pH and lactate exchange via the monocarbocylate transporter system, making it a highly‐promising approach for translation.^33^, ^34^ Blocking the initial steps of excessive ROS production, rather than trying to offset the entire ROS effects further downstream with antioxidants, could be a much more effective approach taking the dual effects of ROS into account. In a similar way, targeting complex I, using the mitochondrial‐targeted S‐nitrosothiol MitoSNO, which S‐nitrosates a particular cysteine residue of complex I, prevents RET and reduces I/R injury.^35^ The fact that complex I can be inhibited by metformin can also explain at least part of the protective action of this biguanide.^36^

#### Monoamine oxidases A and B

The monoamine oxidase A (MAO‐A) and B (MAO‐B) flavoenzymes catalyze the degradation of catecholamines and other biogenic amines to produce hydrogen peroxide and are largely considered to be major producers of mitochondrial ROS. Increased MAO activity is associated with high hydrogen peroxide production alongside highly reactive aldehydes and ammonia which act in synergy to impair mitochondrial function in cardiomyocytes.^37^, ^38^ The beneficial effects of MAO‐A and MAO‐B genetic or pharmacological inhibition as potential drug targets have been extensively studied in various pre‐clinical models of acute and chronic heart diseases including IHD,^39^ diabetic cardiomyopathy, or anthracycline‐induced cardiotoxicity.^37^ However, despite the fact that specific and non‐specific inhibitors of both MAO‐A and MAO‐B isotypes have been available in the clinical practice for decades, human data are scarce on their benefit in IHD, revealing mostly mechanistic information,^37^ but not yet the feasibility to treat or to prevent human IHD. Since the expression level of MAO isoforms varies between species and changes with ageing and dietary status,^37^ the assessment of the clinical benefit of their inhibition is complicated in most animal models and may warrant novel test systems better resembling the humans. Notably, MAO inhibitors display side effects such as hypertensive crises thereby limiting their potential use as cardioprotective drugs.^40^ However, additional MAO inhibitory compounds^41^ such as moclobemide and safinamide that are commonly used for the treatment of depression and neurodegenerative disorders, are devoid of classical side effects of MAO inhibition and therefore may have potential therapeutic application in MI.

#### Iron

Mitochondrial iron also contributes to oxidative stress in the heart.^42^ Iron‐dependent ROS production is involved in I/R injury^43^ and its reduction alleviates ischaemic cardiomyopathy.^44^ Reducing mitochondrial iron genetically through cardiac‐specific overexpression of a mitochondrial iron export protein or pharmacologically using a mitochondria‐permeable iron chelator, protects mice against I/R injury.^44^ Nevertheless, these data are yet to be reproduced in large animals or in clinical scenarios.

#### Reactive oxygen species scavengers and antioxidant enzyme systems

Numerous compounds have been developed that target mitochondria and have direct ROS scavenger activity or activate antioxidant enzymes such as superoxide dismutase‐2 (SOD2). These agents have been tested in model systems involving ischaemia with varying success.^45^, ^46^, ^47^ However, in cardiac pathologies, clinical success still has not been achieved. Mitoquinone (MitoQ) is being investigated in a Phase 2 study to improve myocardial function in dilated cardiomyopathy (NCT05410873), but since concerns have been voiced regarding toxicity issues for several mitochondria‐targeted compounds,^47^ and facing the high number of failed antioxidant candidates in IHD, this field may lose momentum.

In summary, despite being an attractive target, alleviating the damage caused by mitochondrial ROS formation in IHD has not been translated into a clinically applicable treatment strategy, therefore, continued research effort is warranted. The ischaemia‐selective approach of SDH inhibition with malonate is certainly a promising target.

### Permeability transition pore and cell death

The classical cardioprotective strategies involving ischaemic conditioning, including both preconditioning and postconditioning, exert their protective effects, at least in part, by preventing PTP opening.^48^, ^49^, ^50^, ^51^, ^52^ Similarly, several drugs, such as generic antioxidants, have demonstrated cardioprotective effects by protecting against PTP opening.^53^

The mitochondrial PTP is a non‐selective protein channel located in the inner mitochondrial membrane. During myocardial ischaemia, PTP remains closed due to the low pH. However, when reperfusion occurs and the pH rapidly corrects, PTP opens, allowing the entry of molecules that induce mitochondrial swelling and ultimately lead to cell death. Targeting PTP has been considered as an ideal strategy for drugs aiming to mitigate the negative effects of reperfusion injury and limit the size of infarction. However, this has proven to be challenging in both experimental and clinical settings, as so far, available drugs target proteins that modulate PTP opening and not the pore itself. For example, the prototypical PTP inhibitor, cyclosporin A (CsA), binds and inhibits the regulatory component cyclophilin D (CypD).^54^

Although Phase 2 clinical trials indicated that CsA was cardioprotective in ST‐elevation MI (STEMI) patients,^18^, ^19^ two larger clinical trials failed to show any protective effect of CsA on either infarct size or clinical outcome^21^, ^22^ (*Table* *1*). The failure to clinically translate CsA protection may relate on the different solvants used,^55^ but also to the fact that CsA does not target directly the PTP. The specificity of CsA for PTP is also questioned, as numerous experimental studies have demonstrated its cardioprotective effects through mechanisms independent of PTP.^56^ Interestingly, multivariate analysis further revealed that patient outcome depends not only on infarct size but also on gender and the presence of comorbidities, suggesting that the design of pre‐clinical cardioprotective strategies should be re‐evaluated, considering other clinical parameters as outcomes rather than solely focusing on infarct size.^57^ The failure of the MITOCARE clinical trial,^23^ assessing the cardioprotective effect of TRO40303 shown to have *in vitro* PTP inhibition effects, further reinforces the need of more specific PTP inhibitors.

Recent studies reported on the PTP structure: a conformational change of F‐ATP synthase and a re‐evaluation of adenine nucleotide translocator.^58^ Consequently, more specific PTP inhibitors have been designed that inhibit PTP opening with cardioprotective effects, independently of CyPD and without affecting F‐ATPase activity.^59^ Importantly, these are cardioprotective in *in vitro* and *ex vivo* mouse models of MI.^59^ Further research is needed to screen more specific PTP inhibitors with improved *in vivo* use.

### Mitochondrial bioenergetics/metabolism

An alternative treatment for HF could be metabolic interventions that re‐establish metabolic flexibility, for example the use of alternative substrates such as ketone bodies to modify fatty acid and glucose metabolism.^60^, ^61^ In agreement, ketone esters have been shown to protect patients with cardiogenic shock.^62^ Several clinical trials are ongoing to investigate the benefit of targeting mitochondrial metabolism in IHD. For example, the HF‐AF ENERGY trial aims to increase levels of nicotinamide adenine dinucleotide (NAD^+^), an important coenzyme of electron transport chain which decreases in response to I/R injury, participating in mitochondrial dysfunction.^63^ This trial investigates the cardioprotective effects of nicotinamide riboside, a precursor of NAD^+^, in IHD patients diagnosed with atrial fibrillation (AF).^64^

Mitochondria, via the enzymes and the intermediates of their energy production machinery, contribute to epigenetic programming. The foundation of this concept has emerged from studies reporting that Kreb's cycle intermediates such as 2‐oxoglutarate (also known as α‐ketoglutarate), succinate and fumarate, can modulate cellular epigenetics by regulating the activity of enzymes involved in DNA (e.g. TET DNA demethylases) or histone lysine methylation (e.g. KDM) with consequences on senescence.^65^, ^66^ Acetyl‐CoA, a central product of the mitochondrial energy production cascade, is also epigenetically active, functioning as one of the main acetyl groups donors for genome‐wide histone acetylation by the histone acetyl transferases.^67^ The relevance of the metabolism‐dependent epigenetic programming in cardiac pathology is evidenced by inhibitors of various classes of histone deacetylates or DNA methylation which exert cardioprotective actions against acute ischaemia and HF.^68^ Intriguingly, cardioprotective effects were also observed in response to acetyl group donors *in vivo*, which caused a genome‐wide increase in histone acetylation.^69^

### Mitochondrial Ca^2+^ handling

Mitochondrial Ca^2+^ overload is one of the main mechanisms for irreversible mitochondrial dysfunction and subsequent death of cardiomyocytes during I/R injury.^70^, ^71^ In contrast, in the failing heart, reduced Ca^2+^ release from the sarcoplasmic reticulum and increased cytosolic Na^+^ impede mitochondrial Ca^2+^ accumulation, leading to oxidative stress.^72^, ^73^ Mitochondrial Ca^2+^ handling is mainly determined by the function of mitochondrial Ca^2+^ transporters, of which the mitochondrial calcium uniporter (MCU) regulates mitochondrial Ca^2+^ uptake, while Ca^2+^ efflux is controlled by the mitochondrial Na^+^/Ca^2+^ exchanger (NCLX) as primary pathway under physiological conditions. However, during ischaemia and the early phase of reperfusion, the NCLX operates in reverse mode^74^ and thus it is still debated whether mitochondrial Ca^2+^ overload is mediated by the MCU, the NCLX or both during these conditions. In this way, a recent study reported that loss of MCU reduces but does not eliminate the increase in mitochondrial Ca^2+^ during ischaemia.^75^

Dysregulation or dysfunction of the MCU,^76^ the porin VDAC2 or NCLX has been associated with various pathological conditions including IHD and HF. Overexpression of the dominant‐negative MCU pore subunit, MCUb, reduces mitochondrial Ca^2+^ uptake and protects against pathological remodelling in a mouse model of I/R.^77^ miR‐181c, on the other hand, has been shown to activate mitochondrial calcium uptake in the ischaemic heart, by regulating MICU1.^78^ However, while a smaller infarct size following I/R was measured in mice with conditional, cardiomyocyte‐specific deletion of MCU,^79^ whole‐body MCU knockout mice were not protected against cardiac I/R.^80^ Further study revealed a compensatory adaptation in MCU knockout heart, with increased CypD phosphorylation leading to increased PTP opening.^81^ This highlights the potential for misleading conclusions from whole‐body transgenic models. In the context of HF, altered expression and composition of MCU subunits or NCLX have been demonstrated in different pathologies including end‐stage HF,^82^ Barth syndrome,^83^, ^84^ cardiac hypertrophy,^85^ and AF associated with metabolic syndrome.^86^

Due to the dysfunctional mitochondrial Ca^2+^ transporters, several therapeutic strategies have been developed to reduce I/R damage or HF, but tested only in pre‐clinical studies so far. In principle, interventions that reduce the rise in mitochondrial Ca^2+^ during I/R also reduce the amount of myocardial injury. Cardiomyocyte‐specific knockout of the MCU or pharmacological inhibition with Ru360 improved post‐ischaemic cardiac function in mice and rats.^79^, ^80^, ^87^ Since Ru360 has limited membrane permeability, new small molecule inhibitors of MCU activity have been developed,^88^ but their effectiveness in the context of post‐ischaemic cardiac function still needs to be tested in detail. However, when NCLX was overexpressed in a heart‐specific manner, the mice were protected from I/R injury.^89^ Recently, TMEM65 was identified as a novel regulator of NCLX‐dependent mitochondrial calcium efflux. A knockdown of *Tmem65* expression in mice promotes mitochondrial Ca^2+^ overload in the heart and impairs cardiac function.^90^ These data support the concept that acute increased NCLX‐dependent Ca^2+^ efflux during I/R reduces mitochondrial Ca^2+^ and may be a viable therapeutic strategy in disease, while long‐term inhibition is detrimental.

In HF, drugs augmenting mitochondrial Ca^2+^ uptake via the MCU or VDAC2, or preventing Ca^2+^ extrusion via the NCLX might improve cardiac function and protect from arrhythmias. In a translational study, the flavonoid kaempferol has been shown to increase mitochondrial Ca^2+^ uptake and reduce AF in a metabolic syndrome mouse model.^86^ On the other hand, inhibiting mitochondrial NCLX with CGP‐37157 increases mitochondrial matrix Ca^2+^ in a guinea pig model of HF, and prevents left ventricular dysfunction and arrhythmias *in vivo*.^91^ Although both compounds increase steady‐state mitochondrial Ca^2+^ concentrations, they have not been tested in humans thus far due to the high active concentrations and the risk of side effects.

Thanks to the recently identified structure of NCLX and MCU, several drug screens have been performed,^92^, ^93^ notably on Food and Drug Administration (FDA)‐approved drugs^94^ in order to improve the potential for clinical translation. Nevertheless, deciphering the alterations of VDAC, NCLX and MCU during the progression of IHD and HF will be required for mitochondrial Ca^2+^ regulation to be considered as a therapeutic option.

### Mitochondrial dynamics

Mitochondrial dynamics refer to the continual processes of fusion, fission, biogenesis, and mitophagy that mitochondria undergo, and which maintains optimal mitochondrial health and function. During ischaemia or exposure to oxidative stress, the balance is tipped toward mitochondrial fission, resulting in more fragmented mitochondria, decreased mitochondrial respiration and sensitizing the heart to injury.^95^ Thus, inhibiting mitochondrial fission is an attractive target to protect the ischaemic and reperfused heart. Nevertheless, evidence suggests that over a long time‐scale, both mitochondrial fusion and fission are required to maintain mitochondrial quality and cardiac health.^96^

Mitochondrial fragmentation and dysfunction have been observed in human myocardium from patients with pressure overload or IHD with preserved or reduced ejection fraction.^97^ Both mitophagy and mitochondrial biogenesis occur during cardiac surgery involving cardiopulmonary bypass, and are believed to mitigate mitochondrial DNA damage.^98^

Pharmacological compounds developed to specifically target mitochondrial fission proteins have proven effective in reducing infarct size in experimental mouse models of myocardial injury. Mitochondrial division inhibitor 1 (Mdivi‐1), a putative dynamin‐related protein 1 (Drp1) inhibitor was the first compound found to limit acute myocardial I/R injury.^95^, ^99^ However, it is a drug with low potency in mammalian cells (IC_50_ 10–50 μM).^100^, ^101^ In‐silico screening was used to identify Drpitor1a, which inhibits Drp1 GTPase activity more potently and reduces myocardial injury (IC_50_ 0.06 μM).^102^, ^103^ A concern with compounds targeting the GTPase activity of Drp1 is their potential off‐target effects on the GTPase domain of other enzymes such as dynamin. The synthetic peptide P110 inhibits Drp1 function via an alternative mechanism and is cardioprotective in rats.^104^ Despite the promising experimental results in small animal models, a pilot study using Mdivi‐1 in a closed‐chest pig acute MI model provided no significant effects of the treatment on infarct size or cardiac function.^105^ However, the results may be explained by inadequate drug dose or delivery. Nanoparticles have been successfully used to improve delivery of Mdivi‐1 to the heart and reduce I/R injury.^106^

Targeting mitochondrial fusion proteins has proven to be more challenging. However, a cell‐permeant minipeptide inhibiting mitofusin 2 (MFN2) was found to increase mitochondrial fusion in cultured fibroblasts,^107^ and several small‐molecule MFN activators have been identified that work in a similar manner. However, to date, these have mainly been tested in the nervous system, and not in the heart.

It should be noted that most drugs or interventions that increase mitochondrial respiration will also result indirectly in increased mitochondrial biogenesis and fusion.^108^ For example, known therapeutic compounds with cardioprotective properties, such as the sodium–glucose cotransporter 2 inhibitors empagliflozin^109^ and dapaglifozin^110^ also increase mitochondrial fusion.

As yet there are no drugs to specifically stimulate mitochondrial biogenesis, though some activate pathways such as AMP‐activated protein kinase (AMPK) and peroxisome proliferator‐activated receptor gamma coactivator 1‐alpha (PGC‐1α), which are involved in mitochondrial biogenesis. For example, metformin improves left ventricular function and survival in HF via AMPK.^111^ Metformin, resveratrol and acetylcholine protect the heart from I/R injury via AMPK or PGC‐1α.^112^, ^113^, ^114^, ^115^

### Mitophagy and heterophagy

Damaged mitochondria undergo a precise selective elimination through autophagy‐driven lysosomal degradation. This process—termed mitophagy—is essential for cardiomyocyte homeostasis and when perturbed, is broadly associated with cardiovascular disease including IHD and HF.^116^

The two major routes of mitophagy are either ubiquitin‐dependent or receptor‐mediated (ubiquitin‐independent),^117^ while both are mediated through light chain 3 (LC3)‐decorated autophagosomes. When they fail or are inhibited, cells can employ several alternative mitophagy mechanisms. These include LC3‐independent autophagosomes that originate from the trans‐Golgi network, endosome‐mediated degradation, or lysosomal elimination of damaged mitochondria. The latter is also known as micromitophagy and may also play a role in the oxidative stress response upon cardiac I/R.^118^ Damaged mitochondria are either taken up by lysosomes directly and deposited into multi‐organellar lysosomal‐like structures for elimination, or vesicles with damaged content bud off from mitochondria and form multivesicular bodies which are then taken up by lysosomes. Finally, heterophagy describes the heart‐specific mechanism of secreting damaged mitochondria from cardiomyocytes within large membranous vesicles, which are subsequently phagocytosed and degraded by cardiac resident macrophages.^119^

Particularly in the context of cardiac I/R, there is ample pre‐clinical evidence (mainly studies in rodents) for a cardioprotective role of mitophagy.^120^ Therefore, enhancing mitophagy in a preventative manner by food supplements^121^ or therapeutically by the delivery of tissue‐protective factors, present exciting opportunities.^122^ Compounds of interest include NAD^+^ precursors such as nicotinamide, nicotinamide riboside, nicotinamide mononucleotide,^63^ urolithin A^123^ and spermidine.^121^ Nevertheless, targeting mitophagy is at an early stage of development, and it can be difficult to disentangle the effect of drugs affecting mitophagy from other effects they may have on mitochondrial metabolism. Furthermore, caution is warranted as there is also evidence that over‐activated mitophagy can exacerbate myocardial damage.^124^ In addition, the different types of conventional and alternative mitophagy along with the distinct cardiac stresses (I/R, pressure overload, chronic HF, pathogenic gene variant, etc.) highlight that potential mitophagy‐promoting therapies are highly context‐dependent and more translational research is needed.

## Improving the clinical translation of mitochondria‐targeted treatment: main road blocks and opportunities

While our understanding of mitochondrial alterations during IHD and our knowledge on the structure and regulation of key mitochondrial targets have greatly improved over the last decade, no mitochondria‐targeted treatment has shown a cardioprotective benefit for the patients so far. Numerous challenges need to be overcome: timing of drug administration, tissue bioavailability and specificity of mitochondrial targeting, together with the mitochondrial impact of risk factors, comorbidities and co‐medications.

### Challenge in translation: testing the therapy in clinically relevant models

#### Impact of comorbidities, risk factors and co‐medications

Patients with IHD often suffer from comorbidities (diabetes, hypertension, obesity, cancer, etc.), are on co‐medications (anti‐diabetic drugs, anti‐hypertensives, cancer treatment, etc.) and are elderly. Each of these factors can affect mitochondrial functions and may abrogate mitoprotection.^25^, ^125^, ^126^ Alterations in mitochondrial function are recognized as significant contributors to cardiac senescence during ageing. Aged cardiomyocytes exhibit abnormalities in mitochondrial structure and together with increased ROS generation, contributing to left ventricular dysfunction and adverse remodelling.^127^, ^128^

Type 2 diabetes (T2D) and obesity are key comorbidities frequently encountered in IHD patients. Most cardiac mitochondrial functions are impaired in T2D and thus may counteract any mitoprotective strategies.^129^ Similarly, several co‐medications such as anti‐diabetic and lipid‐lowering drugs alter mitochondrial functions, as well as the cardiotoxicity induced by chemotherapeutic agents.

Interestingly, a sexual dimorphism has been reported for several mitochondrial functions (PTP, oxidative stress and respiratory functions),^130^ accounting for sex differences in the response to I/R injury, exemplified by a greater myocardial salvage in STEMI women.^131^

In sum, more research is needed to clearly define the interplay between pre‐existing conditions affecting mitochondria, and mitoprotective treatments in order to proceed towards a personalized medicine in cardiovascular disease.

#### Improving the design of pre‐clinical and clinical studies of mitoprotection

Since comorbidities and medications may counteract or even abrogate mitoprotection, pre‐clinical models should be revisited by conducting studies with aged animals and with the comorbidities that are most prevalent in IHD patients (see IMPACT criteria^132^). Pre‐clinical experimental models of cardioprotection have also been developed to better reflect clinically relevant background therapies of STEMI patients.^133^ Moreover, as in clinical trials, final endpoints should not only focus on infarct size but on long‐term effect, such as improvement of cardiac function and remodelling, and development of HF. Use of large animals, such as pigs,^134^ should also be preferred as there are strong species differences (e.g. heart rate between rodents and humans) and drug bioavailability may differ between pre‐clinical models and clinical settings.

An intermediary step towards investigating the effectiveness of new mitochondrial‐targeted treatments in humans, should include testing the drugs in *in vitro* and *ex vivo* human model systems including patient‐induced pluripotent stem cell‐derived models (e.g. cardiac organoids or engineered heart muscle),^135^ explanted human atrial muscle subjected to hypoxia and reoxygenation,^136^, ^137^ or primary ventricular human heart tissue that can be kept in culture in form of living myocardial slices.^138^ Notably, in addition to the advantage of being human, explanted myocardium also reflects the age, sex, and possibly comorbidities of genuine patients.^139^

Regarding the design of clinical trials, important considerations include the timing of treatment administration and the patient selection – for example whether to include patients with cardiogenic shock or in Killip class III–IV. In order to determine when a patient should be treated with a mitochondrial therapy and if the treatment effectively acts on mitochondria, assessing mitochondrial function in patients will be an invaluable asset, either through cardiac biopsies, measurement of cellular energy status using ^31^P magnetic resonance imaging or circulating biomarkers (lactate/pyruvate ratio, fibroblast growth factor 21).^140^

### Challenge in drug delivery: recent advancements in mitochondrial targeting of drug

Enhancing the ability of drugs to target mitochondria is essential to improve potency, avoid side effects and accelerate the delivery.^140^

#### Transporters and nanocarriers

In order to efficiently deliver biologically relevant cargo molecules to mitochondria, different strategies for achieving mitochondrial accumulation have been developed.^53^ One strategy has taken advantage of the substantial electrochemical potential maintained across the inner mitochondrial membrane and led to the development of a class of molecules, named delocalized lipophilic cations (DLCs). DLCs are particularly effective at crossing the hydrophobic membranes and, being positively charged, preferentially accumulate in mitochondria.^141^ A series of antioxidants have been conjugated to the DLC: triphenylphosphonium (TPP) and derivatives (MTP‐131 [Bendavia, elamipretide]) are such nanocarriers, designed to selectively accumulate in the (negatively charged) inner mitochondrial membrane.^35^, ^142^, ^143^, ^144^, ^145^, ^146^, ^147^, ^148^, ^149^, ^150^, ^151^, ^152^ Of these, the TPP‐ubiquinone conjugate, MitoQ, has gained considerable attention as an antioxidant compound, and pre‐clinical studies have demonstrated that ROS scavenging properties with MitoQ have cardioprotective effects in animal models of pressure overload‐induced HF and I/R injury.^153^ However, as described above these effects were not translated to the clinic.

Polyethylene glycol (PEG) conjugated poly(lactic‐co‐glycolic) acid (PLGA) nanoparticles, a U.S. FDA‐approved copolymer, targets mitochondria through the enhanced permeability and retention effect that is characteristic of injured myocardial tissue.^154^, ^155^, ^156^, ^157^, ^158^ The addition of mitochondriotropic compounds such as SS31 and arginyl‐glycyl‐aspartic acid to the nitric oxide formulation further improves organelle‐ and cell‐specific uptake, respectively.^53^ Combinations of polymeric and lipidic nanoparticles have been developed for the mitochondria to internalize bioactive compounds through endocytosis.^159^, ^160^ Finally, liposome‐based nanocarriers, e.g. the Mito‐porter, have been designed for mitochondrial uptake via macropinocytosis.^161^, ^162^, ^163^, ^164^, ^165^ These nanocarrier‐based delivery strategies were formulated to selectively deliver mitochondria‐targeted cardioprotective molecules notably antioxidants (e.g. coenzyme Q10, MitoQ, 10‐[6'‐plastoquinonyl]‐decyltriphenylphosphonium [SkQ1], Mito‐Tempo).^6^, ^8^, ^142^, ^144^, ^166^, ^167^, ^168^, ^169^, ^170^, ^171^ Nanoformulations allowing for simultaneous drug delivery, as shown by the PLGA nanoparticles combining CsA with pitavastatin to reduce myocardial I/R injury, present exciting novel therapeutic avenues.^155^, ^172^ Despite promising results derived from animal studies, clinical studies will be needed to demonstrate the mitoprotective effect of these agents in the setting of IHD.

#### Mitochondrial peptides

Peptide‐based delivery to mitochondria presents various advantages, including ease and versatility of synthesis, water solubility, biocompatibility, and wide applicability. A major drawback is represented by enzymatic hydrolysis, that can be offset, at least in part, by high concentrations, special modifications, or formulations, potentially causing side effects.^173^ They also tend to be more expensive than small molecules. A group of cell‐permeable small peptides (Szeto–Schiller peptides) that selectively partition to the inner mitochondrial membrane, have been shown to scavenge ROS, reduce mitochondrial ROS production, inhibit mitochondrial PTP opening, reducing apoptosis and necrosis induced by ROS or mitochondrial dysfunction.^174^ Among these, the tetrapeptide elamipretide (MTP‐131) has been shown to interact with cardiolipin and accumulate at the inner mitochondrial membrane, wherein it reduces proton leaks, improves ATP production and ultimately restores mitochondrial function.^175^ In animal models of MI, MTP‐131 improved cardiomyocyte survival during reperfusion, reduced infarct size and the extent of no‐reflow.^176^ However, in subjects with first‐time anterior STEMI undergoing successful percutaneous coronary intervention, MTP‐131 administration did not significantly reduce myocardial infarct size.^6^ In animals with angiotensin II‐mediated cardiomyopathy, MTP‐131 ameliorated cardiac hypertrophy, diastolic dysfunction and fibrosis, despite the absence of blood pressure‐lowering effects.^177^ Recently, in septal myectomy tissues from patients with hypertrophic cardiomyopathy, MTP‐131 improved NADH‐driven mitochondrial respiration.^178^ MTP‐131 also ameliorated left ventricular systolic dysfunction in a pre‐clinical model of advanced HF.^179^ However, although MTP‐131 administration was safe and well tolerated in patients with stable HF with reduced ejection fraction,^9^ it did not improve left ventricular end‐systolic volume or ejection fraction after 4 weeks of treatment.^10^

Mitochondria‐derived peptides (MDPs) are a new class of peptides, which are encoded by small open reading frames within other known genes of the mitochondrial DNA.^180^ MDPs have been shown to play a protective role in myocardial I/R injury. Administration of [Gly14]‐humanin (HNG) 1 h before or at the time of reperfusion reduced infarct size and improved left ventricular function in a mouse model of I/R through AMPK‐endothelial nitric oxide synthase‐mediated signalling and regulating apoptotic factors.^181^ Similarly, other studies showed that high doses of HNG could reduce arrhythmias, myocardial damage area, and mitochondrial dysfunction.^182^ HNG improved mitochondrial damage, by decreasing mitochondrial ROS levels, and was more effective than CsA in alleviating mitochondrial ROS and increasing ATP production.^183^ Modern research techniques, applying nanotechnologies and *in vitro* peptide synthesis, allow greatly increased biologic activity and delivery of MDPs directly to the place of action, thus further expanding their therapeutic application on the cardiovascular system.^184^ Although MDPs might be a new target to treat cardiovascular disease, their effects have been investigated only in experimental systems and further studies are needed to reveal their protective role.

### Challenge in translation: novel strategies

#### A higher level of complexity: mitochondria subtypes in different cell types

The clinical translation of current mitochondria‐targeted strategies is challenged by the existence of specific mitochondrial subtypes in cardiomyocytes.^185^ None of the novel mitochondria‐targeted drugs are studied in relation to the interfibrillar, subsarcolemmal versus perinuclear mitochondria, despite the fact that all subtypes have different morphology, function and response to cardiac pathologies.^51^, ^186^, ^187^

Moreover, mitochondria‐targeted strategies do not consider the differences in mitochondria of different cell types, such as cardiomyocytes, fibroblasts, endothelial cells, leucocytes or platelets. In this context, it has been demonstrated that mitochondrial‐targeted drugs have opposite effects on different cells under the same pathological stimuli.^188^ For example, targeting sirtuin 3 (SIRT3) efficiently increases mitochondrial function and decreases apoptosis in cardiomyocytes,^189^ while it increases mitophagy in cardiac fibroblasts.^190^ Resveratrol, known as a broad SIRT agonist, failed to improve exercise capacity in adults with mitochondrial myopathies,^191^ but exerted cardioprotective effects in experimental animal studies by improving mitochondrial activity in cardiomyocytes.^192^

Mitochondrial‐targeted drugs may also exert protective effects in different cell types. For example, nicotinamide riboside, an NAD precursor in NAD^+^/SIRT1 signalling, prevents blockage of autophagic flux, accumulation of autolysosomes, and oxidative stress in cardiomyocytes,^193^ but also enhances mitochondrial respiration in peripheral blood mononuclear cells of patients with HF, attenuating thereby their proinflammatory activation.^194^ MitoQ improves endothelial function and exercise tolerance in patients with peripheral artery disease,^195^ while also protecting against cardiac hypertrophy in patients with hypertension.^195^ While the studied effects of MitoQ are focused on endothelial mitochondria, the exact mechanisms on other cell types are still unclear. This also accounts for prostaglandin E2 receptor‐4 agonists, such as ONO‐0260164, which efficiently reduce fibrosis in cardiovascular pathologies by acting on cardiac fibroblast's mitochondria, but their effect in cardiomyocytes is still unknown.^196^

Platelet mitochondrial function is also understudied, despite the fact that these are ideal bioenergetic indicators in patients with HF.^197^

#### Mitochondrial transplantation, a controversial mitoprotective strategy

Naked mitochondrial transplantation results in the replacement of injured mitochondria through the exogenous transplantation of isolated intact and functional mitochondria from healthy cells or tissues. In an *in vivo* swine model, temporary coronary occlusion followed by intracoronary mitochondrial transplantation at reperfusion increased coronary blood flow in a mitochondrial concentration‐dependent manner.^198^ Mitochondrial transplantation has been also investigated in HF models, showing that it prevents myocardial apoptosis and boosts myocardial contraction, although the improvement in contraction depends on the metabolic compatibility between transplanted mitochondria and the recipient's cardiomyopathic cardiomyocytes.^199^ Interestingly, mitochondrial transplantation isolated from diabetic cells (donors) shows attenuated cardioprotective effects compared with non‐diabetic mitochondria.^200^, ^201^ To our knowledge, there is only one pilot ‘in‐human protocol’^202^ performed in children (*n* = 5), in which autologous mitochondrial transplantation seemed to improve ventricular function.^203^

The concept of mitochondrial transplantation has been challenged based on a number of claims that are not sufficiently supported by the existing experimental evidence.^204^ First, it is unclear how mitochondria can survive micromolar concentrations of Ca^2+^ when being injected into the coronary blood stream or the heart directly. When simulating these conditions by exposing skeletal muscle mitochondria to micromolar Ca^2+^ concentrations, their membrane potential (ΔΨ_m_) immediately dissipates, and they can no longer respire and do not recover from this Ca^2+^ ‘shock’.^205^ Even if mitochondria survived the Ca^2+^ shock, it is unclear how they can produce ATP when still outside of a cell (as it was proposed), and how this ATP would reach the sites where it is required inside of cells.^204^ Finally, it remains unclear how enough mitochondria can pass the membrane of cells to integrate into the mitochondrial network to eventually contribute to any meaningful additional ATP production inside of cells. Further research is clearly required to resolve these many open questions.^204^, ^206^, ^207^

## Final statement with translational perspectives

Targeting mitochondria in the context of IHD remains a powerful therapeutic strategy to be employed as a combination therapy, as currently envisioned in cardioprotection.^208^, ^209^ The recent technical developments in mitochondrial targeting of drugs, from nanoparticles to peptides via selective activation inside mitochondria, are key opportunities to achieve a better tissue selectivity, through enhanced cardiac and above all, mitochondria targeting, in order to reduce off‐target effects. Mitoprotective strategies could also benefit from current research on mitochondria‐targeted treatment for cancer therapy where hundreds of strategies have been developed. Notably, mitochondria‐targeted free‐drug approach consists in a mitochondria targeting unit coupled to a unit responsible for either aggregation, self‐assembly or polymerization inside the injured mitochondria (based on specific characteristics such as loss of mitochondrial membrane potential).^210^ In order to reach a more personalized medicine for IHD patients, improving our scientific knowledge on the progression of mitochondrial alterations during IHD would be a crucial step to adapt the treatment for each patient. In parallel, all the recent discoveries of structure and regulation of key mitochondrial players during IHD will help to propose specific modulators of these mitochondrial functions, in part thanks to high‐throughput screening of FDA‐approved libraries.

### Funding

Agence Nationale de la Recherche (ANR‐ 20‐CE14‐0013‐01) and Fondation de France (00107048) to MePa. German Research Foundation (Deutsche Forschungsgemeinschaft, DFG) (Project number 471241922; and research training group 2824) and the German Centre for Cardiovascular Research, (DZHK), partner site Göttingen to KSB. AM is supported by the Swiss National Science Foundation (grant number 215035), the Swiss Heart Foundation (project number FF19110) and the Fondation Pierre Mercier pour la Science. Fondation pour la Recherche Médicale (‘Equipe FRM 2021, EQU202103012601’) to F.L. PNRR‐MAD‐2022‐12 375 790 (Italian Ministry of Health) and 12 845‐kfshrc‐2023‐KFSHR‐R‐3‐1‐HW from RDIA granting scheme (Kingdom of Saudi Arabia) to M. Pesce. M.A. received funding from the Austrian Science Fund (FWF; I6931) under the umbrella of the Partnership Fostering a European Research Area for Health (ERA4Health) (GA No. 101 095 426 of the EU Horizon Europe Research and Innovation Programme). M.A. also acknowledges additional support from the FWF (P34926), BioTechMed‐Graz (Young Researcher Group) and Medical University of Graz (Flagship Project VASC‐HEALTH). D.J.H. is supported by the Duke‐NUS Signature Research Programme funded by the Ministry of Health, Singapore Ministry of Health's National Medical Research Council under its Singapore Translational Research Investigator Award (MOH‐STaR21jun‐0003), Centre Grant scheme (NMRC CG21APR1006), and Collaborative Centre Grant scheme (NMRC/CG21APRC006). S.V.L. is supported by the Deutsche Forschungsgemeinschaft (DFG, German Research Foundation), SFB‐1470‐A07. P.K. is supported by the German Research Foundation (RTG 2989). S.M.D. was supported by grants from the British Heart Foundation (PG/21/10798, PG/19/51/34493, PG/16/85/32471). C.G.T. is supported by the Italian Ministry of Health (PNRR‐MAD‐2022‐12 376 632). C.M. is supported by German Research Foundation (DFG; project # 453989101 and # 505805397). C.P. is supported by the Italian Ministry of Health (PNRR‐ MR1‐2022‐12 376 858) and Italian Ministry of University and Research (2020YRETTX_003; CN00000041; Progetti di Rilevante Interesse Nazionale 2022ZPS49L). BJJMB is supported by the Dutch Heart Foundation (2020‐2020B003, DnAFix), NWO (NWA.1389.20.157 CIRCULAR and ERA4HEALTHcvd‐87). This article is based upon work supported by the CArdiovascular DiseasE National Collaborative Enterprise (CADENCE) National Clinical Translational Program, the PREVENT‐HF Industry Alignment Fund Pre‐Positioning Programme (IAF‐PP H23J2a0033), National Research Foundation Competitive Research Program (NRF CRP25‐2020RS‐0001) and COST Actions EU‐CARDIOPROTECTION IG16225 and EU‐METAHEART CA22169 (MePa, PK, IA) supported by COST (European Cooperation in Science and Technology).

**Conflict of interest**: C.B. reports consultant fees from Cardior Pharmaceutical GmbH, outside the submitted work. D.J.H. has received consultant fees from Faraday Pharmaceuticals Inc. and Boehringer Ingelheim International GmbH; honoraria from Servier; and research funding from AstraZeneca, Merck Sharp & Dohme Corp and Novo Nordisk. A.M. reports consultant fees from BioMarin Pharmaceutical Inc, outside the submitted work. K.S.B. received research support from Novartis and BioNtech and speaker's honoraria from Novartis outside this work. C.G.T. received honoraria or consultation fees from VivaLyfe, Univers Formazione, Solaris, Summeet, Myocardial Solutions, AstraZeneca, and Medtronic, outside of the submitted work; funding from Amgen and MSD for clinical trials, and is listed as an inventor of 2 patents related to heart failure. T.K. is founder and Chief Medical Officer of Camoxis Pharmaceuticals. All other authors have nothing to disclose.
